# Pregnancy and COVID‐19: Do not overlook malaria

**DOI:** 10.1002/ijgo.13670

**Published:** 2021-03-29

**Authors:** Marta Papaccio, Roberta Castellani, Cristina Zanardini, Enrico Sartori, Federico Prefumo, Barbara Saccani

**Affiliations:** ^1^ Division of Obstetrics and Gynecology ASST Spedali Civili Department of Clinical and Experimental Sciences University of Brescia Brescia Italy; ^2^ Department of Infectious and Tropical Diseases ASST Spedali Civili Brescia Italy

**Keywords:** chills, chloroquine, COVID‐19, fever, malaria, myalgia, plasmodium ovale, pregnancy

## Abstract

COVID‐19 and malaria may have a similar presentation in pregnancy.

The synchronous co‐infection of malaria and SARS‐CoV‐2 in pregnancy has recently been reported.^1^ A 38‐year‐old woman born in Burkina Faso, gravida 4 para 3, lived in Italy for 8 years until November 2019 when she returned to Burkina Faso for a short visit. She was referred to us in March 2020 at 22^+2 ^weeks of gestation with a two‐day history of fever (38.4°C), dry cough, rhinitis, malaise, myalgia, retrosternal pain, and fatigue. Nasopharyngeal swab SARS‐CoV‐2 RT‐PCR resulted positive, chest X‐ray was unremarkable; however, lung ultrasound was consistent with viral pneumonia. Due to tachypnea (36 breaths/minute) and worsening SpO_2_, the patient started oxygen therapy. Laboratory findings were normal, except for increased levels of C‐reactive protein. Clinical conditions and imaging improved. On the 20th day she was discharged, and nasopharyngeal swabs on days 21 and 22 were negative. No ethical approval was required for this study and the patient provided written informed consent for inclusion in the study.

At 25^+6 ^weeks of gestation she was admitted again with fever (39°C), chills, and myalgia. Chest X‐ray and SARS‐CoV‐2 swab were normal. Blood examination revealed non‐*falciparum* trophozoites; other findings were unremarkable, except for once again increased levels of C‐reactive protein. Empirical therapy with chloroquine (10 mg/kg/day) was started. Peripheral blood smear, parasite nucleic acid detection, and search for malarial specific antigens revealed *P. ovale* infection, hence chloroquine was continued for 3 days. After treatment, blood smear became negative for plasmodia. Follow‐up instructions were given and postpartum eradication with primaquine was planned. The remainder of her pregnancy was uneventful, and she delivered a healthy baby weighing 2664 g at 41^+0 ^weeks of gestation.

SARS‐CoV‐2 infection causes immune system impairment that leads to higher vulnerability to other infections and reactivation of latent infections.^2^ *P. ovale* infection was diagnosed 5 months after her journey to Burkina Faso. Since the incubation period of malaria ranges between 10 and 17 days, primary malaria infection was excluded, and the final diagnosis was a relapse of *P. ovale* infection. This is in accordance with a reported median of 17 weeks (range 2–60 weeks) between primary infection and first relapse of *P. ovale*.^3^ The primary infection was presumably asymptomatic.^4^ SARS‐CoV‐2 infection occurred in March, which could have facilitated *P. ovale* resurgence by impairing the woman's immune response.

In the present study, the patient's presentation was similar in the two admissions, and SARS‐CoV‐2 infection was the initial diagnostic hypothesis on both occasions. Many febrile diseases can mimic COVID‐19, and SARS‐CoV‐2 infection itself may facilitate the onset or reactivation of infectious diseases. In the middle of the COVID‐19 pandemic, we should not overlook other infectious diseases with similar clinical presentations.

## CONFLICTS OF INTEREST

The authors have no conflicts of interest.

## AUTHOR CONTRIBUTIONS

FP and BS conceived and designed this study. MP, RC, CZ and BS contributed substantially to the acquisition of the data. MP, RC, CZ, ES, FP and BS contributed to the interpretation of the results and drafted the paper. MP, RC, CZ, ES, FP and BS revised and approved of the final version of the manuscript.
